# Targeted Protein Degradation to Overcome Resistance in Cancer Therapies: PROTAC and N-Degron Pathway

**DOI:** 10.3390/biomedicines10092100

**Published:** 2022-08-27

**Authors:** Hanbyeol Kim, Jeongbae Park, Jeong-Mok Kim

**Affiliations:** 1 Department of Life Science, College of Natural Sciences, Hanyang University, Seoul 04763, Korea; 2 Research Institute for Natural Sciences, Hanyang University, Seoul 04763, Korea; 3 Hanyang Institute of Bioscience and Biotechnology, Hanyang University, Seoul 04763, Korea

**Keywords:** targeted cancer therapy, PROTAC, N-degron

## Abstract

Extensive progress in understanding the molecular mechanisms of cancer growth and proliferation has led to the remarkable development of drugs that target cancer-driving molecules. Most target molecules are proteins such as kinases and kinase-associated receptors, which have enzymatic activities needed for the signaling cascades of cells. The small molecule inhibitors for these target molecules greatly improved therapeutic efficacy and lowered the systemic toxicity in cancer therapies. However, long-term and high-dosage treatment of small inhibitors for cancer has produced other obstacles, such as resistance to inhibitors. Among recent approaches to overcoming drug resistance to cancers, targeted protein degradation (TPD) such as proteolysis-targeting chimera (PROTAC) technology adopts a distinct mechanism of action by which a target protein is destroyed through the cellular proteolytic system, such as the ubiquitin–proteasome system or autophagy. Here, we review the currently developed PROTACs as the representative TPD molecules for cancer therapy and the N-degrons of the N-degron pathways as the potential TPD ligands.

## 1. Introduction

Cancer is one of the leading causes of death worldwide [1]. Surgery, radiation therapy, and chemotherapy have been commonly used for cancer treatments. However, surgery and radiation therapy are ineffective for metastatic cancer, and chemotherapy often induces systemic cytotoxicity because of non-selectivity between normal cells and cancer cells [2,3]. Immunotherapy, as an alternative method, shows effective cancer treatment with reduced systemic toxicity by targeting cancer through the body’s natural defense system, but it is limited to a subset of cancer types and a minority of patients with such cancers [4,5].

With the progress of research on cancer at the molecular level and technology for drug discovery and delivery, small molecules or monoclonal antibodies that specifically disrupt the function of individual oncogenic drivers, i.e., mainly proteins, have been developed, leading to the renaissance of cancer therapies, called molecule-targeted therapies [6,7,8,9]. The drug used for molecule-targeted therapy is often a chemical like that for chemotherapy which is beneficial for metastatic cancer treatment, but specifically targets the protein which shows abnormal overexpression or has an aberrant function in the oncogenic pathway of a cancer cell [10]. Therefore, it can distinguish a cancer cell from normal cells, which gives higher potency and lower toxicity of molecule-targeted therapy than chemotherapy [10]. Molecular-targeted therapy can be applied to a broad range of cancer types, because it depends on the available drug and its target molecule for a specific type of cancer cell but not on the complicated immune system, as immunotherapy does.

Contrary to its effectiveness for cancer, this revolutionary therapy has faced unpredicted problems such as side effects and drug resistance, at least in monotherapies, demanding alternative strategies against them [9]. As is similar with those of conventional chemotherapy, the side effects of molecule-targeted therapy are caused by the dysregulation of the cellular pathway containing the target molecule upon drug treatment in normal tissues [11]. The target molecule can moderately express and exert its function in a normal cell. The molecule-targeted drug inhibits this molecule, leading to aberrant activation or inhibition of a specific pathway related to drug-induced complications (on-target effect) [12,13]. For example, an epidermal growth factor receptor (EGFR) inhibitor such as gefitinib sometimes causes skin complications such as acneiform eruption, rash, or folliculitis [11]. A vascular endothelial growth factor (VEGF) inhibitor such as bevacizumab leads to hemorrhage, proteinuria, gastrointestinal perforation, and hypertension [14]. Other EGFR inhibitors such as sorafenib and sunitinib inhibit not only their target kinases but also multiple kinases that are unintended target molecules, leading to side effects such as erythema, edema, and hyperkeratosis (off-target effect) [13,15].

The major concern of molecule-targeted therapy for cancer is drug resistance, which is a common obstacle of chemotherapy [10]. Primary or intrinsic resistances might be generated during the tumorigenic process in the pretreatment phase [16]. The cancer cell with primary resistance mostly shows a lack of target dependency in the context of tumor heterogeneity, including insensitive target variant, aberrant pathway linking to the target, and mutation activating downstream of the target [8]. Secondary or acquired resistances often occur after some time from the clinical use of the molecule-targeted drug. Due to secondary resistances, many patients showing promising efficacy after drug treatment fail to achieve complete responses [17]. The main cause of secondary resistance to the molecule-targeted drug might be due to compensatory reprogramming of cancer cells to recover their oncogenic potential as before treatment, but it is still elusive [16]. Instead, several mechanisms of secondary resistance to drug treatment have been elucidated. The bypass pathway activation is the resistant mechanism related to the activation of the downstream or connecting pathway of oncogenic target pathways such as the PI3K pathway, MAPK pathway, or STAT/JAK pathway [18,19]. The cancer cell with this resistance can sustain oncogenic signaling with continuous inhibition of target molecules, such as EGFR and HER2 amplification upon MET exon 14 inhibition in non-small lung cancer (NSCLC) [19,20]. The lineage plasticity is a secondary resistance mechanism related to phenotypic switching, such as epithelial–mesenchymal transition (EMT), through which a cancer cell has increased migration, invasive potential, and resistance to the drug without alteration of its genotype [8,21,22]. It has been reported that NSCLC patient cells acquiring resistance to gefitinib or osimertinib show the EMT features [23]. The on-target alteration is the most directly effective among secondary resistant mechanisms of cancer cells because it renders the target itself through secondary mutation, amplification, or alternative splicing, leading to interfering with the drug–target physical connection [24,25,26,27,28].

Although many strategies with the combination of targeted drugs have been in trials and new targets or new drugs have been developed for overcoming resistances [10], there are still potential problems, because many small molecules for targeted therapy are inhibitors of oncogenic proteins. The small molecule can work as an inhibitor only when it is tightly occupying the pocket, often an enzymatic active site, on a protein. This occupancy-driven pharmacological model requires high drug administration, which possibly increases off-target binding and side effects [29,30,31,32]. Unfortunately, most proteins, up to 75%, including non-enzymatic proteins, transcription factors, and scaffolding proteins, are “undruggable”, which means that they do not have therapeutically effective small molecules [33]. Much worse, after treatment with inhibitors, cancer cells frequently acquire secondary mutations within the target protein, rendering the interface of the active pocket to reduce drug affinity (e.g., Ibrutinib-resistant C481S mutation of Bruton tyrosine kinase (BTK) in chronic lymphocytic leukemia (CLL) [34,35,36,37,38] and osimertinib-resistant C797S mutation of EGFR in NSCLC [39,40,41,42]). The inhibition of a target protein for periods may also lead cancer cells developing alternative signaling pathways or overexpressing the target protein to evade targeted therapy. For example, breakpoint cluster region-Abelson (BCR-ABL) tyrosine kinase and EGFR are overexpressed in imatinib-resistant chronic myeloid leukemia (CML) and crizotinib-resistant anaplastic lymphoma kinase (ALK)-positive NSCLC, respectively [43,44,45]. Monoclonal antibodies (mAbs) might be one of the alternative methods for cancer therapy [46,47], given that mAbs bind specifically to a target protein located on cancer cells but independently of its active pocket, and some mAbs work through different mechanisms to inhibit immune checkpoints such as cytotoxic T-lymphocyte-associated antigen 4 (CTLA-4) or programmed cell death 1 (PD-1) [2]. However, as mAbs also play their roles through protein–protein interaction and/or inhibition, mAb-based cancer therapies also are not completely free of resistance due to secondary mutation or the emergence of alternative signaling pathways, such as small-molecule-based ones [48,49,50].

Recently, targeted protein degradation (TPD) technology has emerged as a new approach for molecule-targeted therapy [51,52,53,54]. The molecule from TPD technology (TPD molecule) mediates target protein degradation through cellular protein degradation systems such as the ubiquitin–proteasome system (UPS) or autophagy [51,54]. Among TPD molecules, proteolysis-targeting chimera (PROTAC) is the major class of molecules that was first developed and has been most extensively studied [51]. It has also motivated the development of other TPD molecules, such as autophagy-targeting chimeras (AUTACs) [55], lysosome-targeting chimeras (LYTACs) [56], and antibody-based PROTACs (AbTACs) [57]. More importantly, PROTAC targets a protein in an event-driven manner, fundamentally different from the classical small molecule inhibitor that does so in an occupancy-driven manner [51]. Because of this prominent feature, PROTACs are attracting attention as an alternative strategy for overcoming resistance to molecule-targeted cancer therapies.

In this review, we briefly summarize the general mechanism, advantages, and current status of PROTACs to overcome resistance to molecule-targeted cancer therapies. We also review the resistance to PROTACs and their derived challenges and propose possible alternative PROTACs which have the potential to be developed in the near future.

## 2. General Mechanism and Advantages of PROTAC

### 2.1. General Mechanism of PROTAC

**Figure 1:**
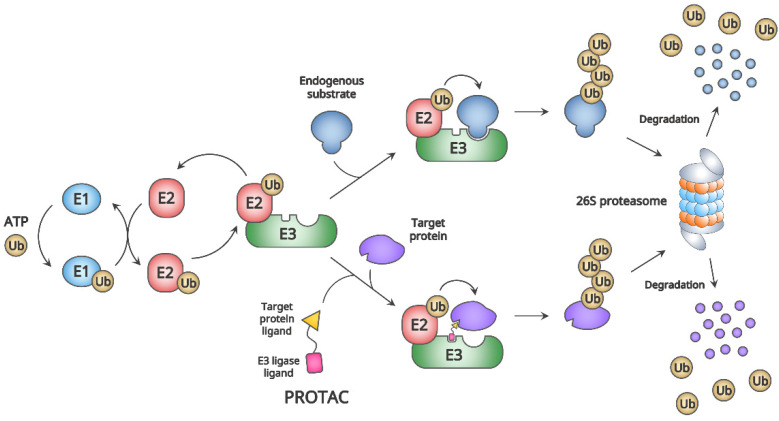
The ubiquitin–proteasome system (UPS) and proteolysis-targeting chimera (PROTAC). For the degradation of a protein through UPS, the ubiquitin (Ub) is linked to an Ub-activating enzyme (E1) via a thioester bond in the ATP-dependent reaction. Next, activated Ub on E1 is transferred to an Ub-conjugating enzyme (E2) through trans-thioesterification. The Ub-ligase (E3) recognizes a degradation signal (degron) of an endogenous substrate and conjugates a Ub from E2 to a lysine residue of the substrate protein. The serial cascades of Ub-activation, Ub-conjugation, and Ub-ligation by E1, E2, and E3 polyubiquitylate the substrate or substrate-conjugated Ub. The 26S proteasome recognizes the polyubiquitin chains on the substrate, subsequently followed by deubiquitylation, unfolding, and degradation of the substrate. The PROTAC can hijack the E3 by its ligand and recruit a target protein for degradation through UPS (adapted from [54,58]).

The PROTAC is a heterobifunctional molecule that functions via UPS for the degradation of a target protein and is composed of two different ligands fused by a linker, in which one ligand binds a target protein and the other recruits an E3 ubiquitin ligase (E3 ligase) [51,54]. The UPS is the universal proteolytic system for protein homeostasis, including quality control to eliminate aberrant proteins in eukaryotic cells [54]. It comprises a set of processes for proteolysis, in which the main components are ubiquitin (Ub), Ub-activating enzyme (E1), Ub-conjugating enzyme (E2), E3 ligase, and 26S proteasome (Figure 1). Briefly, the Ub is joined to an E1 with a thioester bond through an ATP-dependent reaction, and Ub is conjugated to an E2 from the E1 via trans-thioesterification. The E3 ligase, as the complex with Ub-E2, recognizes a target protein through its degradation signal (degron) and conjugates Ub to a residue (usually a lysine) of the target protein. This cyclic cascade is repeated to further ubiquitylation on other residues or already-attached ubiquitin on the target protein, yielding a polyubiquitylated one. The 26S proteasome unfolds and degrades the target protein via the recognition of poly-Ub chains [58]. By hijacking this proteolytic pathway, the PROTAC targets a protein for degradation (Figure 1).

### 2.2. Advantages of PROTAC

Different from classical small molecule inhibitors that must occupy the active pocket of a target protein for inhibition (occupancy-driven mechanism), PROTACs do not have to necessarily bind the active pocket of a target protein for degradation because they initiate the degradation event of a target protein (event-driven mechanism) [51,54]. In detail, one equivalent PROTAC molecule brings an equivalent of the target protein to the zone of ubiquitylation structurally defined by the catalytically active site of the recruited E3 ligase. After polyubiquitylation and subsequent degradation of the target protein, the already used but remaining PROTAC molecule repeatedly can bring another equivalent of the target protein into the E3 ligase domain [54,59,60,61]. Thus, PROTAC in low concentrations might be sufficiently more effective to achieve therapeutic goals and lower off-target effects than classical inhibitors.

As mentioned above, PROTAC does not require the active pocket of a target protein for its function. It also does not require a high affinity for a target protein [51]. These two features, due to the event-driven mechanism of PROTAC, allow it to target a variety of proteins. In other words, PROTAC can theoretically target any protein, whether it is “druggable” or “undruggable”, unless there is no ligand for that protein. Even in the absence of a ligand for the target protein, developing PROTAC might not be difficult because the action of PROTAC is not limited to the small area on the target protein, like a small molecule inhibitor [30,31]. Given that many proteins exist as multi-subunit complexes in the cell [62,63,64], and PROTAC recruits E3 ligase in proximity to the target protein [51], PROTAC for the subunit near the target protein in the complex might be another strategy for degradation of the target protein.

The molecule-targeted therapy by small molecule inhibitors often has met resistance due to secondary mutation in the active pocket of the target protein, compensatory overexpression of the target protein or downstream signaling proteins, or partial inhibition of a multi-domain protein containing a scaffolding domain [8,9,65]. Because PROTAC mediates the degradation of a target protein differently from inhibition by a small molecule inhibitor, PROTAC can solve these resistances through downregulation of the target protein (including its drug-resistant mutant) and subsequent prevention of downstream signaling without restriction [54,66,67].

## 3. Small Molecule PROTACs for Molecule-Targeted Cancer Therapies

The concept of PROTAC was successfully proven through an experiment using the first PROTAC (Protac-1) developed in 2001 [68]. It comprised a 10-amino-acid IkBα phosphopeptide, recruiting β-transducin repeat-containing E3 ubiquitin-protein ligase (β-TRCP), and ovalicin, a small molecule inhibiting methionine aminopeptidase 2 (MetAP2). Protac-1 could target MetAP2 for β-TRCP-dependent polyubiquitylation, followed by the degradation of MetAP2 in Xenopus egg extracts [68]. Although Protac-1 showed a proof-of-concept for PROTAC, it could not be directly applied as a strategy for drug development because it had low cell permeability due to its high molecular weight and metabolically unstable peptide bonds owing to many intracellular or extracellular peptidases [69]. However, small molecule ligands for several E3 ligases such as MDM2 [70], cellular inhibitor of apoptosis protein 1 (cIAP1) [71,72,73], cereblon (CRBN) [74,75], and Von Hippel-Lindau (VHL) [76,77] have been reported and they have accelerated the development of PROTAC technology as a novel strategy for molecule-targeted therapies, possibly substituted for those with classical small molecule inhibitors. However, CRBN- or VHL-based PROTACs have become dominant because of their well-characterized physicochemical properties, specificities, and small sizes [53].

### 3.1. CRBN-Based PROTACs

Thalidomide and its immunomodulatory imide drug (IMiD), such as pomalidomide, inhibit the activity of CUL4-RBX1-DDB1-CRBN (CRL4CRBN) E3 ligase by binding to the substrate recognition component CRBN in human cells [78,79,80,81,82]. The first developed CRBN-based PROTAC is dBET1, which is composed of pomalidomide recruiting CRBN and JQ1-inhibiting BRD family members [83]. It has been reported that dBET1 target for degradation BRD2, BRD3, and BRD4 [83]. The CRBN-based PROTAC DT-6 has been recently developed and it effectively reduces the intracellular levels of TGF-β1 [84], which is crucial for tumor progression and immune escape [85,86]. Additionally, many other PROTACs have already been developed or are being developed based on IMiDs recruiting CRBN to target cancer-related proteins, including CDK6, MCL-1/BCL-2, BCL-xL, HDAC6, BTK, BRAF, FKBP12, PARP1, BCR-ABL, FAK, and PD-L1 [66,67,69,87,88,89].

### 3.2. VHL-Based PROTACs

VHL is the substrate recognition component of the multi-subunit E3 ligase complex comprising Elongin B, Elongin C, Cullin-2, Rbx1, and VHL [90], and is the tumor suppressor of clear cell renal cell carcinoma (ccRCC) [91,92]. The E3 ligase complex with VHL can target hypoxia-inducible factor α (HIF) for degradation [93]. As small ligands for the HIF-binding site on VHL had been identified, these ligands were engaged to generate VHL-based PROTACs such as PROTAC-ERRα (for ERRα), PROTAC-RIPK2 (for RIPK2), ARV-771 (for BRD4), CP5V (for Cdc20), and DT2216 (for BCL-xL) [60,94,95,96]. The PROTAC pioneer Crews group has continuously developed a series of VHL-based PROTACs targeting BCR-ABL in CML [66]. It has been reported that ARD-61 with the androgen receptor (AR) ligand ARI-16 showed effective degradation of AR in metastatic castration-resistant prostate cancer (mCRPC) [97].

### 3.3. MDM2-Based or IAP-Based PROTACs

MDM2, as an E3 ligase, can bind to p53 and subsequently decrease its level [98,99]. The first MDM-based PROTAC was the linked molecule between selective androgen receptor modulator (SARM) targeting AR and nutlin-3a binding to MDM2 followed by the disruption of the MDM2-p53 complex [100]. Although nutlin-3a has a high binding affinity for MDM2, few MDM2-based PROTACs have been developed, except BRD4-targeting A1874 [101] and PARP1-targeting compound 3 [102]. 

The first cIAP1-based PROTAC was developed with cIAP-ligand methyl bestatin (MeBS) and all-trans retinoic acid (ATRA) for targeting cellular retinoic acid binding protein (CRABP-I and II) [71,72]. Different from other PROTACs, IAP-based PROTACs degrade both the target protein and IAP, requiring a careful design of IAP-based PROTACs [103].

## 4. Resistance to PROTACs and Remaining Challenges

In the molecule-targeted therapies through TPD with PROTACs, the most crucial component is E3 ligase, which mediates polyubiquitylation and subsequent proteasome-dependent degradation of the target proteins [54,66,89]. Although more than 600 E3 ligases are estimated to exist in human cells, nearly all PROTACs recruit an E3 ligase CRBN, VHL, MDM2, or IAP [104,105]. As mentioned above, in the field of PROTACs, CRBN and VHL are the most frequently recruited E3 ligases [53]. A PROTAC follows an event-driven mechanism and thus it has the basic potential to overcome resistant mechanisms to therapeutic agents such as small molecule inhibitors [51,66]. However, cancer cells can rapidly develop resistance to new drugs, and preclinical studies using CRBN- or VHL-based PROTACs have revealed that this concern arrives in the real world [106,107,108]. Either CRBN-based ARV-825-resistant or VHL-based ARV-771-resistant OVCAR-8 cells have shown mutations and/or downregulation of corresponding E3 ligase machinery rather than those of the target protein [106]. Studies in acute myeloid leukemia cell lines have also acquired nearly the same results [107]. Coinciding with these resistances, multiple myeloma patients treated with IMiDs, also used for PROTACs as E3 ligase ligands, have acquired genomic mutations in the components of the CRBN ligase machinery [109]. The resistant mechanism against PROTACs is probably E3-ligase-specific, given that either a VHL-based or CRBN-based one exerted its activity in cell lines resistant to the CRBN-based or VHL-based one, respectively [106]. This phenomenon might give us a key solution to overcoming PROTAC-derived resistance by identifying and developing alternative E3 ligases for PROTAC as a new player for cancer therapy.

## 5. Expansion of PROTACs beyond CRBN and VHL E3 Ligases

### 5.1. Possible Alternative E3 Ligases for Novel PROTACs

Recent efforts to expand the scope of the PROTAC have made somewhat significant progress in the development of PROTACs based on alternative E3 ligases. The aryl hydrocarbon receptor (AhR) is an E3 ligase with a dual function as a transcription factor related to the regulation of cellular responses [110]. The AhR ligand β-naphthoflavone (β-NF) conjugated with CRABP2 ligand ATRA could induce the degradation of CRABP2 [111]. The Kelch-like ECH-associated protein-1 (KEAP1) E3 ligase is involved in the regulation of oxidative stress response through interaction with nuclear factor erythroid 2-related factor-2 (Nrf2) [112]. The PROTACs based on a reversible covalent ligand (methyl bardoxolone) [113] and a non-covalent ligand (MS83) [114] for KEAP1 accelerated the degradation of BRD4. The DCAF15 E3 ligase function for suppression of natural-killer-cell-mediated cancer cell clearance. The sulfonamide E7820-based PROTACs induced DCAF15-dependent degradation of BRD2, BRD3, and BRD4 [115]. The chloroacetamide-bearing compound (TRH 1-23) was conjugated to JQ1 and then recruited RNF4 E3 ligase involved in spermatogenesis for degradation of BRD4 [116]. The natural product nimbolide [117] and non-natural product EN219 [118] could bind to the same site of RNF114 E3 ligase. The PROTACs based on these products degraded BRD4 in an RNF114-dependent manner. The EN106-bearing chloroacetamide could covalently bind to the FEM1B E3 ligase that degrades FNIP1 as the reductive stress response. The JQ1-EN106 conjugated PROTAC was shown to induce degradation of BRD4 [119]. The putative nuclear E3 ligase DCAF16 ligand KB02 was also shown to be able to degrade BRD4 when coupled with JQ1 [120]. The 21-SLF compound was screened as a ligand for DCAF11 E3 ligase which degrades a cell cycle checkpoint protein p21. Conjugated with the androgen receptor ligand (ARL), the compound 21-SLF degraded the androgen receptor mediated by DCAF11 [121]. As novel chemical technologies and structure-guided drug design approaches are developed, the E3 ligase toolbox for PROTACs will distinctly be expanded [122]. However, continuous validation and characterization for alternative E3 ligases and their ligands are first needed, because all of them are still at the starting point compared to CRBN-based or VHL-based PROTACs, each of which has well-defined physicochemical features.

### 5.2. Non-Small Molecule PROTACs (NSM-PROTACs)

The development and proliferation of cancer cells are closely related to signaling oncogenic pathways, and most signaling pathways have receptors located on the cell membrane. The key components for immunotherapy are also extracellular or membrane proteins. The PROTAC is an effective way to reduce oncogenic targets through degradation, but its action is restricted to the area within the cell [123].

The lysosome-targeting chimera (LYTAC) has recently emerged, and it can induce the degradation of target molecules on the cell membrane through the endosome–lysosome pathway [56,124]. The LYTAC molecule is composed of a small molecule or antibody for the target membrane protein and conjugated with a ligand for lysosome-targeting receptors (LTRs) such as cation-independent mannose-6-phosphate receptor (CI-MPR) or asialoglycoprotein receptor (ASGPR) [56,124]. The formation of a ternary complex among the LYTAC molecule, the target membrane protein, and LTR leads to internalization of the target via clathrin-mediated endocytosis, subsequently followed by degradation [123]. The LYTAC molecule conjugated with cetuximab, which is the EGFR antibody, successfully degrades EGFR localized on the cell membrane [56]. When combined with the anti-PD-L1 antibody, the LYTAC molecule also could significantly degrade the PD-L1 [56]. In addition, the liver-specific ASGPR-based LYTAC molecule could target for degradation liver-specific membrane proteins [124]. 

The DNA aptamer also can be used for the degradation of the target protein on cell membranes [125]. The bispecific aptamer chimera, which is composed of DNA aptamer, could mediate the lysosomal degradation of membrane proteins such as receptor tyrosine kinase MET and PTK-7 [125]. Although the bispecific aptamer chimera operates in a similar way to LYTAC, DNA aptamers might have several advantages, such as stability and preparation, compared to antibody-based LYTAC molecules [123]. 

Another TPD technology targeting the membrane protein is the antibody-based PROTAC (AbTAC). It is composed of bispecific antibodies in which one arm binds to the target and the other arm recruits the transmembrane E3 ligase, RNF43 [126]. The AbTAC molecule also degrades the target membrane protein similar to the LYTAC molecule via the endosome–lysosome pathway [57]. However, AbTAC is not yet a promising technology because the mechanism of action is less clear, and RNF43 is the only available receptor for an AbTAC molecule [123].

Recently developed GlueTAC is also based on lysosome-dependent degradation, but it is comprised of a nanobody for a target molecule on the cell membrane, and cell-penetrating peptide (CPP) and lysosome-sorting sequence (LSS) for facilitating internalization and lysosomal degradation [127,128]. In addition, GlueTAC adopts unnatural amino acids in a nanobody for covalent interaction between nanobody and antigen. Experimentally, the GlueTAC molecule is effective in decreasing PD-L1 levels and inhibiting tumor growth in immunodeficient mice more than atezolizumab, an FDA-approved PD-L1 antibody [129]. However, the safety of the introduction of unnatural amino acids in the GlueTAC molecule and the formation of covalent bonds between nanobody and antigen are not yet verified [123].

## 6. N-degron Pathways as a Possible Novel Strategy for PROTAC

The N-degron pathways are a set of proteolytic systems in UPS. The N-degron pathways recognize proteins containing the N-terminal (Nt-) degradation signals, called N-degrons, through the N-degron recognition components, called N-recognins [130]. These recognition events cause the degradation of target proteins by proteasome or autophagy [131,132,133,134]. The N-recognins are either specific E3 ligases or other proteins that target N-degrons [130]. All 20 amino acids, depending on the cognate sequence context, can be a destabilizing Nt-residue, which is the main determinant of an N-degron [130]. Thus, many proteins and their protease-cleaved C-terminal fragments in cells are potential substrates for the N-degron pathways. The targeted degradation by the N-degron pathways is involved in a variety of cellular processes, including the elimination of misfolded or abnormal proteins, sensing of oxygen, quality control of subunit stoichiometries in protein complexes, regulation of DNA repair and replication, and regulation of G proteins, gluconeogenesis, and fat metabolism [58,135,136,137,138]. The N-degron pathways are divided into at least four branches based on each corresponding N-degron, Arg/N-degron [139,140], Ac/N-degron [141,142], Pro/N-degron [143,144], and fMet/N-degron [145,146].

**Figure 2:**
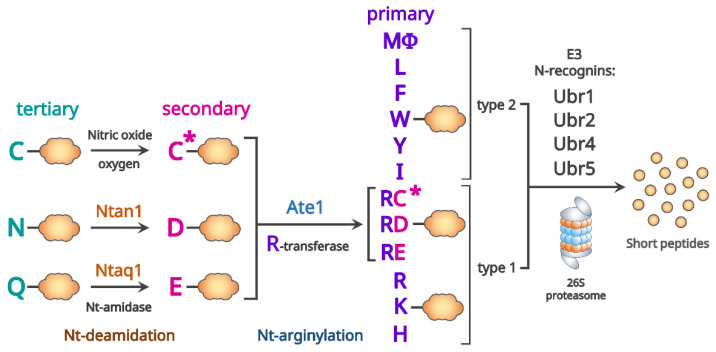
The mammalian Arg/N-degron pathway. In mammals, Ubr1, Ubr2, Ubr4, and Ubr5 E3 ligases are N-recognins of the Arg/N-degron pathway. Arg/N-recognins have at least two binding sites each for type 1 or type 2 primary destabilizing Nt-residues. The tertiary N-terminal (Nt-) destabilizing residues are N-terminally deamidated (Nt-deamidation) by Nt-amidase, Ntan1 for Nt-Asn residue, and Ntaq1 for Nt-Gln residue, respectively. The Nt-Cys residue is exceptional; when Nt-Cys residue is oxidized (denoted by C*), it becomes the secondary Nt-destabilizing residue (but not always). The secondary Nt-destabilizing residues are Nt-arginylated by Arg-tRNA-transferase Ate1. Amino acid residues are denoted by single-letter abbreviations, and the rest of a substrate protein is denoted by an orange cloud (adapted from [130,148]).

The Arg/N-degron pathway (Figure 2) targets for degradation a substrate protein with unmodified Nt-residues recognized by Arg/N-recognins [140,147]. In mammals, Ubr1, Ubr2, Ubr4, and Ubr5 E3 ligases are Arg/N-recognins [58,132,136]. The primary destabilizing Nt-residues (Arg, Lys, His, Leu, Phe, Tyr, Trp, and Ile) are directly recognized by Arg/N-recognins, but secondary destabilizing Nt-residues (Asp and Glu) and tertiary destabilizing Nt-residues (Asn and Gln) are recognized by Arg/N-recognins after preceding Nt-arginylation by Ate1 and Nt-deamidation by Ntan1 or Ntan1, respectively [148,149].

Recently, two profound compounds, ArgT^ERRa^ and His-^TERRa^, have been reported [150]. They are bifunctional molecules, one of which comprises an Nt-Arg-bearing adaptor molecule and a ligand for ERRα, but the other has an adaptor molecule bearing Nt-His instead of Nt-Arg. Either compound effectively reduced endogenous ERRα levels by degradation and decreased the proliferation and migration of MCF-7 metastatic breast cancer cells, demonstrating N-degron-based PROTACs as a possible novel strategy [148,150].

Another fascinating N-degron-based PROTAC has also been developed [151]. This PROTAC is composed of an N-degron motif and a selective steroid receptor co-activator-1 (SRC-1) binding peptide. The aberrantly elevated expression or increased activity of SRC-1 is detected in many types of cancers, strongly implicating SRC-1 in metastasis, drug resistance, recurrence, and poor prognosis [152,153]. Extensive studies have proven that the PROTAC, N-recognin E3 ligase, and SRC-1 form a ternary complex capable of targeted degradation and that SRC-1 is specifically downregulated among other SRC family member proteins [149,151]. The PROTAC-mediated degradation of SRC-1 elicited the downregulation of colony stimulating factor-1 (CSF-1) mRNA and upregulation of E-cadherin mRNA, both of which are known SRC-1 target genes [149,151]. Remarkably, PROTAC targeting SRC-1 decreased MDA-MB-231 cell invasion without affecting viability and reduced lung tumor metastasis in a mouse model, strongly suggesting the therapeutic potential of N-degron-based PROTACs [149,151].

Given that the N-degron pathways have a broad spectrum for substrate proteins in cells [130], N-degron-based PROTACs might be an alternative strategy to overcome drug resistance due to traditional inhibitors or currently developed PROTACs. Moreover, the N-degron-based PROTACs might have tactical advantages of selective or combinatory operations, because each branch of the N-degron pathway, such as the Arg/N-degron pathway, Ac/N-degron pathway, Pro/N-degron pathway, or fMet/N-degron pathway, has its own N-degron and corresponding N-recognin.

## 7. Conclusions

The concept and efficacy of molecule-targeted therapies are innovative compared to traditional cancer therapies such as surgery, chemotherapy, and/or radiotherapy. However, the strategies using small molecule inhibitors have several limitations. First, they always need the activity pocket of a target protein, which only ~25% of human proteins have. Second, a small molecule inhibitor can show adequate efficacy for therapy only when its multiple equivalents occupy almost all the active pockets of multiple equivalents of the target protein in cells. It often requires either high affinity for the target or high-dosage administration of the drug. Third, these two limitations of small inhibitors leave many cancer-related proteins as “undruggable” targets. Fourth, long-term or high-dosage treatment of small inhibitors could lead to toxic side effects, including off-target effects, or give a chance to cancer cells to develop drug-resistant mechanisms such as secondary mutation, compensatory overexpression, or bypass of the target in the signaling cascade. Among these limitations, the worst one might be drug resistance, if there is no alternative to cancer therapy.

Fortunately, PROTAC technology has emerged and then accelerated after the identification of small ligands for E3 ligases. PROTAC targets the protein for degradation via its bifunctional module, recruiting both the target protein and E3 ligase in proximity. Because PROTAC mediates for the target protein the degradation event via UPS in cells, that is, it works in an event-driven manner, PROTAC requires neither the active site nor high affinity for it. The event-driven mechanism of PROTAC has been proven in many preclinical studies as a successful alternative strategy to overcome resistance to targeted cancer therapies due to the occupancy-driven mechanism of small molecule inhibitors. PROTAC technology can also expand the range of targets for therapy by forcing the “undruggable” ones into the druggable region. However, cancer cells can develop resistant mechanisms to PROTACs through mutation and/or downregulation of E3 ligase machinery corresponding to CRBN or VHL E3 ligase, based on which most PROTACs have been designed.

The intensive efforts expanding the scope of PROTAC modality have succeeded in finding possible alternative E3 ligases such as AhR, KEAP1, DCAF15, RNF4, RNF114, FEM1B, DCAF11, and DCAF16. However, their physicochemical features are not yet understood and not validated for them to become genuine alternatives to CRBN and VHL, as the basement of PROTAC technology. It is hard for the PROTAC to degrade the extracellular and membrane proteins often crucial for immune disease and cancer. The non-small molecule PROTACs such as LYTAC, AbTAC, bispecific aptamer chimera, and GlueTAC could be good complements for the treatment of membrane proteins, but they also have to be sufficiently validated for clinical use.

Recently developed N-degron-based PROTACs, which utilize the N-degron pathways for target degradation, are now in the proof-of-concept stage. Although not yet fully validated, N-degron-based PROTACs may become one of the prominent PROTACs not far from now, given the properties of the N-degron suitable for drug development, such as diversity, small size, and chemically simple structure.

The C-degron pathway is a eukaryotic degradation pathway for proteins found in 2018 [154]. Contrary to N-degron, the C-degron is present at the C-terminus of a protein. The C-degron pathways comprises 2-4 amino acids with a few conserved motifs, and glycine is frequently at the C-terminus [155]. A target protein bearing C-degron is degraded by CRL2 or CRL4 E3 ligases [156]. The CRBN-based and VHL-based PROTACs also mediate the degradation of a target protein by CRL2 and CRL4, respectively. Depending on C-degron such as Gly/C-, RxxG/C-, and Arg/C-degron, the substrate recognition component of CRL2 or CRL4 is interchangeable with other components. For example, KLHDC2, KLHDC3, or KLHDC10 of CRL2 recognizes Gly/C-degron, and APPBP2 of CRL2 recognizes RxxG/C-degron. TRPC4AP and DCAF12 of CRL4 recognize the R-3 and E-2 motif, respectively [155,156]. The VHL is a substrate recognition component of CRL2, and CRBN is that of CRL4. Thus, C-degron and its cognate component might be explored for the development of alternative PROTACs to VHL- or CRBN-based PROTACs.

Although PROTAC has achieved many advances in terms of technology, there are still some challenges to be addressed.

First, a few E3 ligases among ~600 E3 ligases are still utilized for PROTAC-mediated target degradation. It depends on the availability of the ligand molecule that binds or recruits a specific E3 ligase. Although new ligands for E3 ligases are found or synthesized with the progress of chemical synthetic techniques and screening methods, more advances are needed for rapid screening of well-designed ligands for an E3 ligase, considering the emergence of PROTAC-resistant cancers.

Second, the ligand molecule for the target protein could restrict the applicable range of PROTAC for cancer therapy. The PROTAC can degrade any target molecule if a ligand is available. However, many of the target ligands conjugated with PROTAC are derived from conventional drugs that bind “druggable” target molecules. To overcome this limitation, designing and screening of a ligand for a target molecule should be performed, at least, in parallel with the development of a ligand for an E3 ligase. Given that a ligand for an E3 ligase can be combined with various ligands for target molecules, the development of target ligands has to be much faster than that of E3 ligands.

Third, the linker between both ligands could restrict the therapeutic effect of a PROTAC molecule. The target protein with a PROTAC is degraded only when it lays in the ubiquitylable zone of an E3 ligase. Therefore, a non-optimized linker of a PROTAC could lead to the failure of cancer therapy. A linker optimized to a PROTAC might disturb the degradation of a target protein in the other PROTAC depending on the E3 ligand or target ligand. It cannot be accurately predicted without structure determination for a ternary complex of E3, target, and PROTAC. The preparation of various linkers with different lengths, charges, or conformations might be helpful but does not guarantee a successful combination of a PROTAC.

Fourth, there is not yet an evaluation system for PROTAC. Without exact evaluation, we cannot use a PROTAC in the clinical setting because the therapeutic efficacy and cytotoxic effect of a PROTAC are unclear, thus having potential risk. Much more studies and efforts are urgently needed to establish the evaluation system for PROTAC.

Nevertheless, the PROTAC would be one of the convincing strategies for cancer therapy, given that it has distinct advantages over conventional drugs for cancer. The challenges for PROTAC might be solved in the near future, together with the development of computer-aided technologies for the production, prediction, and validation of newborn PROTACs.
